# Network-based disease fingerprinting with neuroinflammation PET imaging

**DOI:** 10.21203/rs.3.rs-7887022/v1

**Published:** 2025-10-21

**Authors:** Leonardo Barzon, Lucia Maccioni, Michelle Carranza Mellana, Julia J. Schubert, Ludovica Brusaferri, Oliver Cousins, Ivana Rosenzweig, Yuya Mizuno, Tiago Reis Marques, Neil A. Harrison, Tim Fryer, Edward T. Bullmore, Valeria Mondelli, Carmine Pariante, David Sharp, Gregory Scott, Joana B. Pereira, Oliver Howes, Vesna Sossi, Benedetta Bodini, Bruno Stankoff, Marco L. Loggia, Federico E. Turkheimer, Mattia Veronese

**Affiliations:** Department of Information Engineering, University of Padova, Padova, Italy; Department of Information Engineering, University of Padova, Padova, Italy; Paris Brain Institute, ICM, CNRS, Inserm, Sorbonne Université, Paris, France; Institute of Psychiatry, Psychology and Neuroscience (IoPPN), King’s College London, London, UK; Computer Science and Informatics, School of Engineering, London South Bank University, London, UK; Institute of Psychiatry, Psychology and Neuroscience (IoPPN), King’s College London, London, UK; Institute of Psychiatry, Psychology and Neuroscience (IoPPN), King’s College London, London, UK; Institute of Psychiatry, Psychology and Neuroscience (IoPPN), King’s College London, London, UK; Psychiatric Imaging Group, MRC London Institute of Medical Sciences (LMS), Hammersmith Hospital, Imperial College London, London, UK; Cardiff University Brain Research Imaging Centre (CUBRIC), Cardiff University, Cardiff, UK; Department of Clinical Neurosciences, School of Clinical Medicine, University of Cambridge, Cambridge, UK; Department of Psychiatry, School of Clinical Medicine, University of Cambridge, Cambridge, UK; Institute of Psychiatry, Psychology and Neuroscience (IoPPN), King’s College London, London, UK; Institute of Psychiatry, Psychology and Neuroscience (IoPPN), King’s College London, London, UK; Division of Brain Sciences, Department of Medicine, Imperial College London, UK; Division of Brain Sciences, Department of Medicine, Imperial College London, UK; Division of Neuro, Department of Clinical Neuroscience, Karolinska Institute, Stockholm, Sweden; Institute of Psychiatry, Psychology and Neuroscience (IoPPN), King’s College London, London, UK; Department of Physics and Astronomy, University of British Columbia, Vancouver, BC, Canada; Paris Brain Institute, ICM, CNRS, Inserm, Sorbonne Université, Paris, France; Paris Brain Institute, ICM, CNRS, Inserm, Sorbonne Université, Paris, France; Departments of Radiology and Anesthesia, Critical Care and Pain Medicine, Massachusetts General Hospital, Harvard Medical School, Boston, MA, USA; Institute of Psychiatry, Psychology and Neuroscience (IoPPN), King’s College London, London, UK; Department of Information Engineering, University of Padova, Padova, Italy

**Keywords:** Neuroinflammation, PET, TSPO, Inter-regional similarity, Machine Learning

## Abstract

Neuroinflammation is a hallmark of numerous neurodegenerative, psychiatric, and chronic pain disorders and can be assessed in vivo with 18 kDa translocator protein (TSPO) positron emission tomography (PET). However, conventional quantification methods of TSPO PET are limited and often overlook the spatial relationships between regional signals. The application of network-based approaches to TSPO PET imaging may provide a novel framework to capture disease-specific neuroinflammatory patterns. To address this question, here we developed a data-driven, network-based approach to generate individual brain-wide TSPO PET matrices, employing Euclidean distance to quantify inter-regional pharmacokinetics similarity. We applied this approach to a large multicenter dataset of 528 PET scans utilizing three different TSPO tracers ([^11^C]-PBR28, [^18^F]-DPA714, [^11^C]-PK11195), including healthy controls and patients with different diseases such as multiple sclerosis, traumatic brain injury, schizophrenia, depression, and chronic low back pain. Statistical modelling and machine learning classifiers were applied to evaluate the impact of experimental and biological factors on TSPO similarity patterns and to investigate their potential for capturing disease-specific signatures. TSPO similarity patterns demonstrated high biological specificity and reproducibility, with strong test-retest correlations (mean Spearman’s ρ = 0.84). Average precision of disease classification exceeded chance performance by 23–89% across conditions and was driven by condition-specific regional hubs whose topological distributions closely mirrored disease pathophysiology. This specificity was further supported by minimal overlap in feature importance values across conditions. Altogether, our findings show that network-based analysis of human TSPO PET data can detect disease-specific neuroinflammatory signatures. Such methodologies underscore the biological significance of TSPO PET and enhance its translational value, supporting precision medicine strategies for neuroinflammatory disorders.

## Introduction

Neuroinflammation is the immune response of the central nervous system (CNS) to injury, infection, or other challenges to the homeostasis (DiSabato et al., 2016) by microglia, astrocytes, endothelial cells, and infiltrating peripheral immune cells (Kreutzberg, 1996). While neuroinflammation plays a critical neuroprotective role in maintaining CNS homeostasis, particularly in the acute context, sustained or dysregulated activation can lead to detrimental effects and is increasingly recognized as a core pathological mechanism in a growing number of brain disorders. These include traumatic brain injury, multiple sclerosis, Alzheimer’s disease, Parkinson’s disease (Gerhard et al., 2006; Heneka et al., 2015; Schuitemaker et al., 2013; Tournier et al., 2020; Yong, 2022), major depressive disorder, schizophrenia (Aricioglu et al., 2016; Brites & Fernandes, 2015; Doorduin et al., 2009; Müller, 2018; Troubat et al., 2021) and chronic pain syndromes (Loggia, 2024; Loggia et al., 2015; Torrado-Carvajal et al., 2021).

The 18 kDa translocator protein (TSPO) has emerged as a putative biomarker for neuroinflammation due to its elevated expression in activated glial and immune cells, particularly microglia, during CNS immune responses (Kim & Yu, 2015; Malpetti et al., 2024; Wijesinghe et al., 2025). Consequently, TSPO-targeted molecular imaging using positron emission tomography (PET) has become a widely utilized approach for in vivo quantification of neuroinflammatory processes (De Picker & Haarman, 2021; Jain et al., 2020; Werry et al., 2019). However, several methodological challenges have limited the clinical and translational potential of TSPO PET imaging as a biomarker for neuroinflammation (Turkheimer et al., 2015). Quantification using arterial input functions is limited by the invasiveness of arterial blood sampling and the complexity of measuring radioactivity and correcting for metabolites and protein binding (Tonietto et al., 2016), challenges further compounded by the low free plasma fraction of TSPO ligands (Albrecht et al., 2016). The use of ratio metrics(Lammertsma & Hume, 1996; J. Schubert et al., 2021) has demonstrated promise. However, identifying a pseudoreference region often requires substantial validation effort, as TSPO expression, although relatively low, is widespread across most brain cell types (Turkheimer et al., 2007). Additionally, in some cases the use of ratio metrics may reduce sensitivity to effects that are widespread across the brain. Together, these challenges have motivated the development of alternative modelling strategies that reduce invasiveness while maintaining biological specificity (Maccioni et al., 2025). Beyond technical limitations, most TSPO PET studies have traditionally focused on either localised or widespread increases in magnitude of brain TSPO radiotracer binding, thus overlooking possible alterations in the network of inter-regional relationships that could reflect disease-specific neuroinflammatory profiles. Only few studies have examined how inflammation propagates across different brain regions (Gerhard et al., 2000; Radlinska et al., 2009), and systematic analyses of inter-regional TSPO signal similarity are still lacking. Analytical frameworks that capture the spatial organization of TSPO signals could therefore provide deeper insights into neuroinflammatory processes, revealing patterns that might be masked when regions are considered independently and informing more precise disease characterization and targeted therapeutic approaches.

In line with this, network-based methodologies applied to PET imaging appear particularly well-suited (Sala et al., 2023; Veronese et al., 2019). Traditionally based on group-level covariance analyses, the field has increasingly shifted toward new techniques capable of generating individual-level matrices that capture the tracer pharmacokinetics relationship between brain regions. These subject-specific network representations hold promise as biomarkers for disease diagnosis, stratification, and progression monitoring (Liu et al., 2016; Lu et al., 2024; Severino et al., 2025). Recently, Volpi and colleagues introduced a novel approach to assess the coherence of [^18^F]-fluorodeoxyglucose (FDG) PET brain regional time-activity curves (TACs), employing Euclidean distance as an alternative to conventional correlation-based measures (Volpi et al., 2023). This method provides a valuable perspective on regional signal synchrony, potentially offering enhanced sensitivity to subtle network alterations often overlooked by traditional correlational metrics.

In this work, we propose a novel data-driven approach to generate single-subject TSPO similarity matrices that characterize the inter-regional relationships of TSPO PET pharmacokinetics within a network structure. Rather than relying on simple dynamic radioactivity measures, our method exploits a pre-defined set of biologically meaningful parameters that has been shown to summarize the inflammatory status of cerebral tissue (Maccioni et al., 2025), applying a metric derived from Euclidean distance to compute the similarity between regional inflammation.

To validate our framework, we used a large dataset comprising 528 TSPO PET scans acquired with three different radiotracers ([^11^C]-PBR28, [^18^F]-DPA714, [^11^C]-PK11195), across multiple sites and scanners. This dataset included both healthy controls (HCs) and individuals with a broad spectrum of acute and chronic neuroinflammatory conditions: psychiatric disorders associated with a mild inflammatory state (depression, DEP; schizophrenia, SCZ), conditions with acute central inflammation (multiple sclerosis, MS; traumatic brain injury, TBI), and patients with chronic low back pain (cLBP) as a model of inflammation with extra-cerebral origin). To our knowledge, no study has yet applied methodologies that take into account the similarity between different brain regions to human neuroinflammatory data at the single-subject level. Therefore, after evaluating the biological specificity and reproducibility of TSPO similarity matrices, we applied statistical and machine learning techniques to assess their sensitivity to both experimental and biological variables and evaluate their potential for disease fingerprinting or individual subject identification. The overarching hypothesis of this study is that this framework can uncover distinct network patterns reflective of unique inflammatory signatures, potentially distinguishing each condition in a data-driven and clinically meaningful way.

## Results

### TSPO network topology across subjects, scanners, and radiotracers in healthy individuals

**Figure 1.**
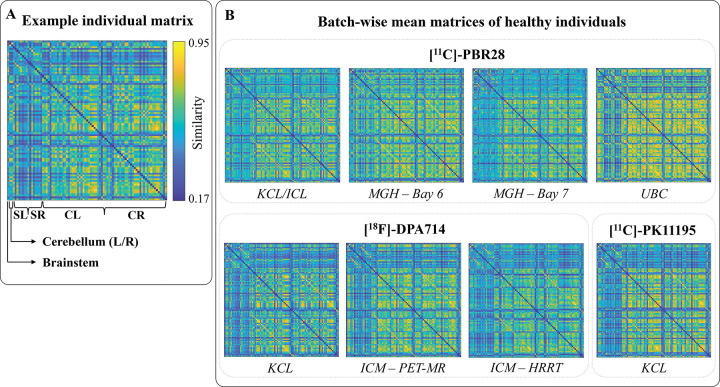
TSPO similarity matrices and cross-centre comparison. Panel A displays a representative similarity matrix from a HC subject, with axis labels showing the organization of ROIs. Panel B shows the average similarity matrices across healthy individuals for each dataset/batch. Acronyms: KCL, King’s College London; ICL, Imperial College London; MGH, Massachusetts General Hospital (Bay 6 and Bay 7 indicate the two scanners); UBC, University of British Columbia; ICM, Paris Brain Institute (PET-MR and HRRT indicate the two scanners); ROI, region of interest; L, left; R, right; CL, cortical left; CR, cortical right; SL, subcortical left; SR, subcortical right.

Single-subject TSPO similarity matrices were computed by calculating the normalized inverse of the Euclidean distance between vectors of regional PET tracer uptake and kinetic parameters, including standardized uptake values at 1.25, 13.5, and 50 minutes post-injection, as well as the tracer blood-to-brain influx rate (*K*_*1*_). Each network is uniquely represented by a matrix, where each element denotes the TSPO pharmacokinetic similarity between two regions of interest (ROIs) (see Materials & Methods for details). An illustrative example of the resulting matrix in a HC is presented in Fig. 1A. These matrices exhibited good inter-subject consistency with comparable network profiles across HC individuals. Across-subjects average similarity matrices by site showed higher similarity among cortical regions than among subcortical regions or cortico-subcortical connections (Fig. 1B). Remarkably, the matrices captured the expected correspondence between homologous cortical, subcortical, and cerebellar regions across hemispheres, as evidenced by prominent secondary diagonals.

**Figure 2.**
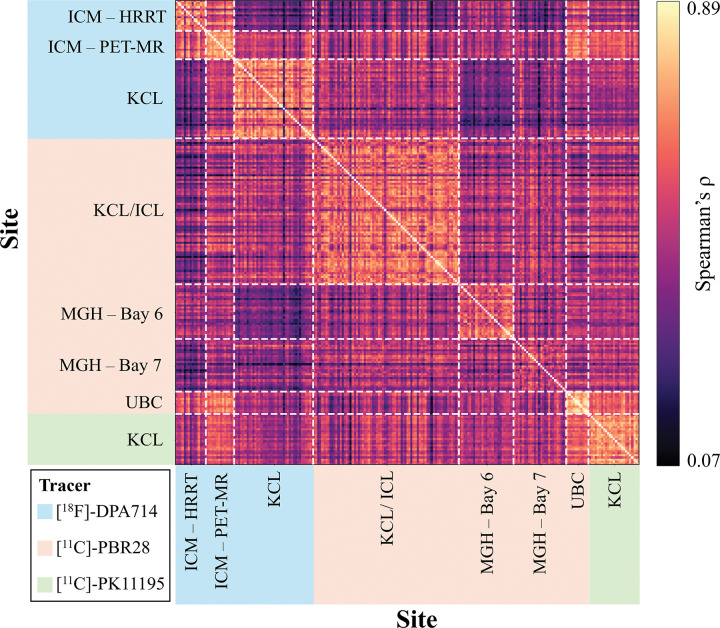
TSPO similarity matrices inter-subject correlations. The figure represents the inter-subject correlations of network patterns, calculated as Spearman’s correlation coefficients between the upper-triangular elements of the TSPO similarity matrices across healthy controls; tracer and site groupings are also indicated. Acronyms: KCL, King’s College London; ICL, Imperial College London; MGH, Massachusetts General Hospital (Bay 6 and Bay 7 indicate the two scanners); UBC, University of British Columbia; ICM, Paris Brain Institute (PET-MR and HRRT indicate the two scanners).

TSPO network patterns appeared generally consistent across different centres. This was reflected by the moderate to high correlations in the inter-subject correlation matrix (Fig. 2), with a global median Spearman’s correlation (ρ ± median absolute deviation) of 0.47 ± 0.09. The highest correlations were typically observed within centres and among scans acquired with the same radiotracers. Specifically, intra-tracer Spearman’s ρ was 0.50 ± 0.13 for [^11^C]-PBR28, 0.53 ± 0.19 for [^18^F]-DPA714, and 0.69 ± 0.07 for [^11^C]-PK11195. Inter-tracer similarity was lower, with values of 0.42 ± 0.12 for [^18^F]-DPA714 vs [^11^C]-PBR28, 0.47 ± 0.10 for [^18^F]-DPA714 vs [^11^C]-PK11195, and 0.49 ± 0.10 for [^11^C]-PBR28 vs [^11^C]-PK11195. Nonetheless, the relatively high inter-subject similarity suggests there were comparable biological patterns across tracers and centres.

### TSPO networks are biologically specific and reproducible

**Figure 3.**
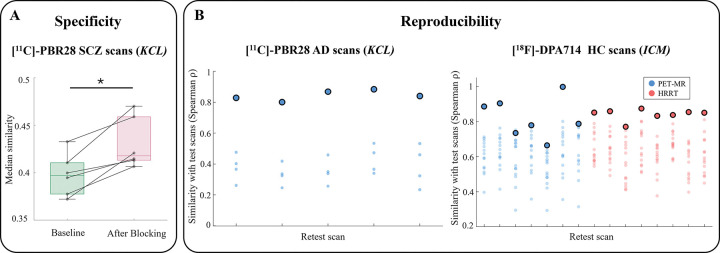
Biological specificity and reproducibility of the network-based methodology. Panel A displays the distribution of median inter-regional similarity values before and after TSPO binding blockade with XBD173 in individuals with SCZ scanned using [^11^C]-PBR28 at *KCL*. A one-tailed Wilcoxon signed-rank test revealed a statistically significant reduction following blockade (* indicates P<0.05). Panel B presents test-retest results from the AD patients scanned with [^11^C]-PBR28 at *KCL*, and the HCs acquired with [^18^F]-DPA714 at *ICM*. Each dot represents the Spearman’s rho between the edge values of a given retest scan and those of all test scans within the corresponding site. The highlighted dot indicates the correlation between the test and retest scans of the same individual. Acronyms: SCZ, schizophrenia; AD, Alzheimer’s disease; KCL, King’s College London; ICM, Paris Brain Institute (PET-MR and HRRT indicate the two scanners).

The biological specificity of the TSPO similarity matrices was assessed by comparing network patterns before and after partial pharmacological blockade of TSPO target with XBD173 injection in a cohort of 6 SCZ patients (Marques et al., 2021; Veronese et al., 2018). As expected, after pharmacological blocking, we observed higher average inter-regional similarity values due to a flatter and more homogeneous distribution of signal across brain regions (Fig. 3A). A one-tailed Wilcoxon signed-rank test confirmed a significant increase in median similarity after blocking (mean percentage increase 8.3%; P = 0.02).

To assess network reproducibility, we used two test-retest datasets: 5 patients with Alzheimer’s disease (AD) scanned twice with [^11^C]-PBR28 (within ~ 12 weeks) and 15 HC subjects scanned twice with [^18^F]-DPA714 (within 5 to 321 days). We observed strong Spearman’s correlations between the upper-triangular values of the test and retest similarity matrices in both datasets (mean ± SD: 0.85 ± 0.03 in AD patients and 0.83 ± 0.08 in HCs). The edge-wise intraclass correlation coefficients (ICC), computed for each matrix element and summarized as median ± median absolute deviation, indicated good reliability in AD patients (0.78 ± 0.14) and moderate reliability in HCs (0.53 ± 0.14). Finally, network fingerprinting potential (J. Liu et al., 2018) was evaluated in terms of its accuracy in identifying, for each test scan, the corresponding retest scan according to the correlation between test and retest TSPO similarity matrices. Accuracy reached 100% in the AD cohort and 93.3% in the HCs cohort, with only one test scan for which the retest scan was the second-most similar rather than the top match (Fig. 3B).

### Effects of technical and biological variability on TSPO similarity matrices

The sensitivity of TSPO similarity matrices to various experimental and biological variables was evaluated in healthy control subjects by investigating possible batch effects due to scanner and acquisition protocol differences, as well as possible effects of age, sex, genotype (specifically the TSPO gene rs6971 polymorphism, which affects the binding affinity of second-generation TSPO radiotracers (Owen et al., 2012)), and radiotracer dose-over-weight ratio (DW). First, machine learning classifiers were applied to test whether we could predict acquisition site, sex, and TSPO binding affinity from individual TSPO similarity matrices (see Materials & Methods for details). The different sites were indeed classified with highest accuracy for both [^11^C]-PBR28 and [^18^F]-DPA714 (balance accuracy > 0.89), while sex and TSPO genotype were classified with moderate (balanced accuracy range: 0.51–0.68 for genotype, 0.63–0.75 for sex), showing variable performance for the three tracers (Table 1).

**Figure 4.**
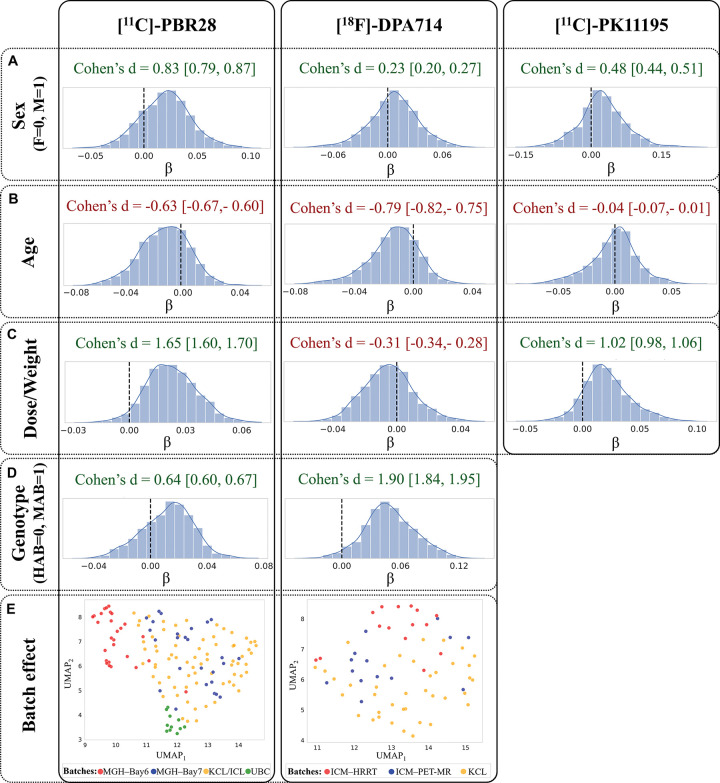
Global effects of demographic and experimental variables on graph edge values in healthy subjects. The first four rows (panels A-D) display the distributions of beta coefficients from linear regression models fitted at each network edge, with sex, genotype, age, and DW as predictors. For each predictor, Cohen’s *d* relative to zero is reported together with 95% confidence intervals, while black dashed lines mark the zero-reference value. The bottom row (Panel E) illustrates batch effects, showing two-dimensional UMAP projections of network edge values. Subjects are colour-coded according to batch membership (KCL: King’s College London; MGH, Massachusetts General Hospital (Bay 6 and Bay 7 indicate the two scanners); UBC, University of British Columbia; ICM, Paris Brain Institute (PET-MR and HRRT indicate the two scanners)).

A linear model was also fitted for each element of the upper triangular matrix (network edge) to quantify the effect of age, sex, binding affinity, and DW across subjects. Model coefficient estimates demonstrated a consistent effect of predictors across tracers as represented by the coherent sign of the Cohen’s *d* of the model coefficients distribution relative to zero (Fig. 4A-D). Specifically, a global higher inter-regional similarity was observed in males compared to females, reflected by positive Cohen’s *d* values (Cohen’s *d* [95% confidence interval (CI)]: 0.83 [0.79, 0.87] for [^11^C]-PBR28, 0.23 [0.20, 0.27] for [^18^F]-DPA714, 0.48 [0.44, 0.51] for [^11^C]-PK11195). Similarly, mixed-affinity binders (MABs) exhibited higher overall similarity compared to high-affinity binders (HABs) (Cohen’s *d* [CI]: 0.64 [0.60, 0.67] for [^11^C]-PBR28, 1.90 [1.84, 1.95] for [^18^F]-DPA714). Conversely, increasing age was associated with reduced global coherence between regions (Cohen’s *d* [CI]: −0.63 [−0.67, −0.60] for [^11^C]-PBR28, −0.79 [−0.82, −0.75] for [^18^F]-DPA714, −0.04 [−0.07, −0.01] for [^11^C]-PK11195). The tracer DW showed positive effects on similarity for [^11^C]-PBR28 (Cohen’s *d* [CI]: 1.65 [1.60, 1.70]) and [^11^C]-PK1195 (Cohen’s *d* [CI]: 1.02 [0.98, 1.06]) tracers, whereas a moderately negative effect was observed for [^18^F]-DPA714 (Cohen’s *d* [CI]: −0.31 [−0.34, −0.28]). The associations were derived after removing the batch effect across all cohorts, which proved to be the strongest covariate, as illustrated by the Uniform Manifold Approximation and Projection (UMAP) plot shown in Fig. 4E.

### Disease classification performance and feature importance

Binary logistic regression models with L1 regularization and stratified 10-fold cross-validation were trained to distinguish HCs from disease cohorts, using elements from the upper triangular portions of TSPO similarity matrices as predictors, after regressing out batch effects and potential confounders related to technical and biological variables. Classification performance, measured by Average Precision (AP) on the test set, consistently exceeded AP chance level—defined as the prevalence of disease-positive subjects in each dataset. Importantly, the lower bound of the 95% confidence interval remained above this threshold in all cases, demonstrating robust and reliable discrimination between patients and controls across conditions (Table 2).

**Figure 5.**
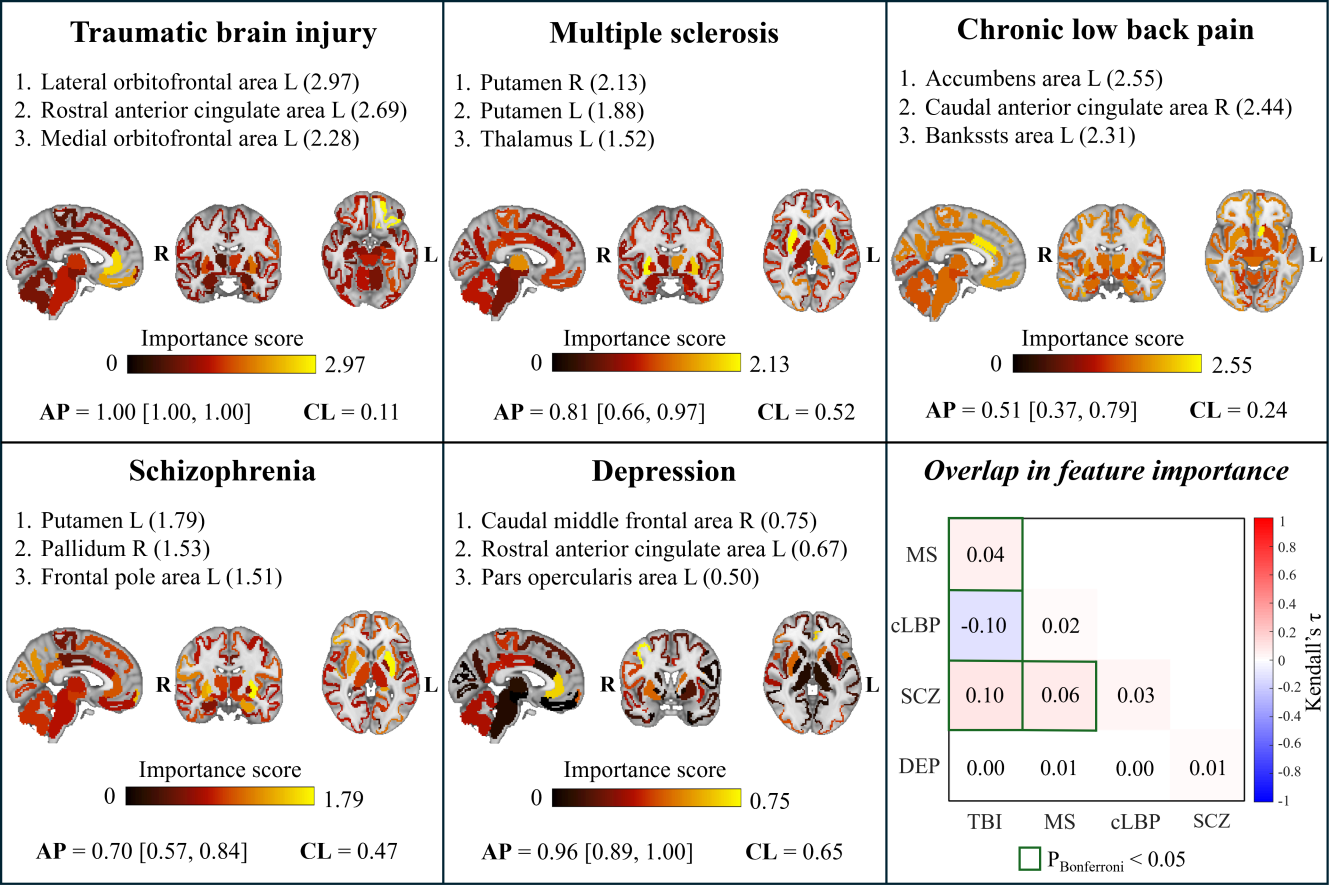
Regional contributions to diagnostic classification. For each condition, brain maps show regional importance scores for disease classification, computed from the absolute values of logistic regression coefficients of network edges connected to each region. For each classifier, the three regions with the highest contributions and their corresponding score values are reported. Classification performance is summarized by the average precision (AP) score on the test set, reported together with the 95% confidence interval and the AP chance level (CL). The box in the bottom right shows the overlap between network edge logistic regression coefficients across conditions, quantified using Kendall correlation. Cells outlined in green indicate correlations that survived Bonferroni correction for multiple comparisons. Acronyms: MS, multiple sclerosis; cLBP, chronic low back pain; SCZ, schizophrenia; DEP, mild-to-moderate depression; TBI, traumatic brain injury; L, left; R, right.

Regional contribution to the classification of each brain anatomical region was quantified by summing the absolute values of the logistic regression coefficients corresponding to the region edges. For every condition, the regions with the highest importance scores were distinct and disease-specific (Fig. 5). The top three contribution regions for each condition, alongside their importance score, were: left lateral orbitofrontal area (2.97), left rostral anterior cingulate area (2.69) and medial orbitofrontal area (2.28) for TBI; right and left putamen (2.13 and 1.88, respectively) and left thalamus (1.52) for MS; left accumbens area (2.55), right caudal anterior cingulate area (2.44) and left bankssts area (2.31) for cLBP; left putamen (1.79), right pallidum (1.53) and left frontal pole area for SCZ; right caudal middle frontal area (0.75), left rostral anterior cingulate area (0.67), and left pars opercularis area (0.50) for DEP. Consistent with disease specificity, Spearman’s correlations between feature importance values (i.e., logistic regression beta coefficients) were generally low across conditions, with a maximum ρ of 0.10 obtained for SCZ vs TBI (Fig. 5, bottom right panel). The top network edges contributing to each disease classification are detailed in **Supplementary Materials S.1**. Additionally, a multiclass logistic regression classifier, trained on data from all tracers and centres, successfully discriminated among the five disease groups (test set balanced accuracy [95% CI]: 0.81 [0.68, 0.90]). The corresponding confusion matrix, including per-group recall values, is provided in **Supplementary Materials S.2**.

### Individual-level patient fingerprinting

**Figure 6.**
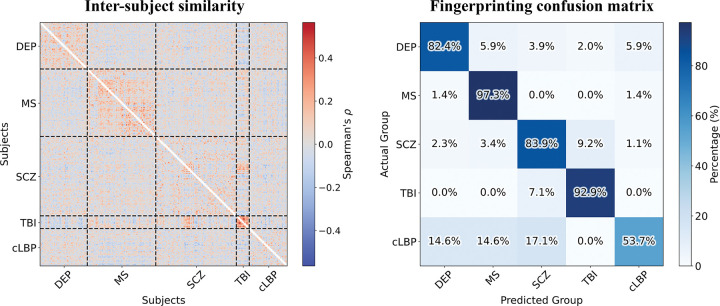
Results of individual patient fingerprinting analysis. The left panel presents the inter-subject similarity matrix for patients, computed using Spearman’s rho. The right panel displays the fingerprinting confusion matrix, summarizing the identification accuracy of individual patients based on their network patterns. Acronyms: DEP, mild-to-moderate depression; MS, multiple sclerosis; SCZ, schizophrenia; TBI, traumatic brain injury; cLBP, chronic low back pain.

Individual-level patient fingerprinting was performed by assigning each patient to the diagnostic group of the subject showing the highest pairwise Spearman correlation between their TSPO similarity matrices, after regressing out batch effects from the graph edges. Results of this analysis are presented in Fig. 6. The inter-subject Spearman correlation matrix revealed distinct blocks of high correlations within diagnostic groups. The same pattern is represented in the fingerprinting confusion matrix, which demonstrates particularly high recall for MS (97.3%), TBI (92.9%), DEP (82.4%), and SCZ (83.9%), whereas a lower recall was observed for cLBP (53.7%). The overall fingerprinting balanced accuracy was 82.0%, exceeding the chance level of 20.0%. When the same analysis was replicated on the single [^11^C]-PBR28 *KCL* dataset, without the necessity to regress out batch effects, the performances of SCZ and TBI patients classification remained above chance level (balanced accuracy: 73.1%; recall: 53.3% for SCZ, 92.9% for TBI).

## Discussion

In this study, we developed a novel analysis framework for TSPO PET, aimed at identifying disease-specific neuroinflammatory fingerprints. We derived TSPO similarity matrices by computing the distance between regional pharmacokinetic parameters, which have been shown to capture TSPO expression profiles (Maccioni et al., 2025). Our approach is conceptually similar to individual-level brain morphological similarity networks (Cai et al., 2023), which are typically constructed by calculating distances between regional magnetic resonance imaging (MRI)-derived features (Sebenius et al., 2023; H. Wang et al., 2016; Yu et al., 2018). Comparable methodologies have also been applied to PET-derived parameters across brain regions to generate individual-specific graphs (Protas et al., 2023; M. Wang et al., 2020; Zhu et al., 2022). However, to our knowledge, our study represents the first application of subject-level network analysis to TSPO PET imaging. Our method was validated on a large multicenter dataset comprising scans acquired across different facilities, using various tracers, PET scanners, and acquisition protocols. Despite this heterogeneity, the resulting individual TSPO PET pharmacokinetics similarity matrices demonstrated good consistency across healthy subjects. However, a clear batch effect was observed, highlighting the importance of accounting for site- and scanner-related variability when analyzing graphs derived from multicenter PET data.

The network-based analysis was performed on a set of cortical and subcortical regions, given the widespread expression of TSPO throughout the brain and its potential to be altered in pathological conditions (Nutma et al., 2021; Wijesinghe et al., 2025). A consistent pattern that emerged from the similarity matrices was a higher degree of similarity among cortical compared to subcortical regions or cortico-subcortical regions similarities. This may reflect a greater uniformity in the distribution and regulatory mechanisms of TSPO across cortical regions, as well as more consistent tracer pharmacokinetic characteristics within the cortex. Additionally, this effect might be partly driven by technical factors such as a higher signal-to-noise ratio of the ROI average TSPO PET signal in cortical areas compared to deep brain structures, which could enhance the detectability of regional relationships. Importantly, the similarity matrices reliably captured the correspondence between homologous regions across the two hemispheres, further supporting a biological interpretation of the identified patterns and the sensitivity of the approach to meaningful inter-regional connections.

Notably, the observed inter-regional similarity pattern was preserved across tracers. This is particularly relevant because, despite the nominally identical target, these radioligands differ in affinity and pharmacokinetic properties (Rizzo et al., 2019). Nevertheless, a well-defined spatial organization was maintained, consistently replicated beyond the technical and experimental differences between studies. Such stability is likely related to the high within-subject reproducibility, enabling near-perfect individual test-retest fingerprinting.

The biological specificity of the proposed approach was validated on pharmacological blocking data, showing a significantly increased inter-regional similarity after target blocking, reflecting a flatter and more homogeneous distribution of signal across brain regions. This is likely due to the predominance of non-specific over specific signal following target occupancy, although the block was not complete (Marques et al., 2021; Veronese et al., 2018), possibly limiting the magnitude of the observed increase.

The ability of TSPO similarity matrices to identify disease-specific signatures was explored across a wide spectrum of neuroinflammatory conditions. These ranged from neuropsychiatric disorders such as DEP and SCZ, to diseases characterized by acute neuroinflammation, such as MS, and TBI, as well as a condition involving peripheral pathology (cLBP). By employing machine learning classifiers, we demonstrated the potential of TSPO PET network-based features to reliably distinguish patients affected by these conditions from healthy controls. Notably, the brain regions contributing most to disease classification closely reflect the known pathophysiological mechanisms of each disorder, highlighting the disease-specific nature of the identified patterns. To support the biological relevance of the data-driven findings, homologous bilateral regions frequently appear among the top contributors to disease classification, underscoring the robustness and value of these results. Remarkably, the emergence of disease-relevant patterns from the network-based analysis may suggest that TSPO PET imaging possesses disease specificity, further reinforcing its biological relevance. However, it is important to acknowledge that these inter-regional similarity patterns could reflect a variety of underlying biological factors, and this study is not equipped to disentangle or attribute them to specific mechanisms. Such patterns may capture a range of processes, including the spreading of activated microglia, structural alterations, or structural and functional disconnections associated with neuroinflammatory states. Therefore, while these network-derived features have demonstrated robust biological relevance, their exact neurobiological basis remains to be fully elucidated. The following disease-specific discussion will further explore these potential interpretations in detail.

Regarding TBI, our study included patients who had experienced moderate-to-severe traumatic events. By nature, this population is heterogeneous, as the type and extent of injury vary according to the trauma mechanism. However, concussions, a significant part of TBI, are often associated with frontal lobe involvement due to the characteristic back-and-forth movement of the brain within the skull (Malloy & Aloia, 1998). Typically, the primary impact initiates a cascade of secondary biochemical processes, including inflammation, predominantly affecting the injury site and adjacent tissues, though neurodegeneration can also extend to distant brain regions (Borlongan et al., 2015). In this group, our analysis revealed prominent involvement of frontal regions, particularly the orbitofrontal cortex, along with subcortical structures such as the putamen. These hubs are highly consistent with the known pathophysiology of TBI, as frontal and subcortical areas are especially susceptible to damage following head trauma. Lesions in these regions have been associated with disruptions in frontal-subcortical circuits, encompassing the orbitofrontal, dorsolateral, and medial frontal cortices, together with the thalamus and striatum, including the putamen (McAllister, 2011).

In MS, our analyses identified the bilateral putamen and thalamus as the principal regional hubs. Notably, putaminal atrophy has been consistently reported in MS, beginning shortly after the onset of clinical symptoms and, in some cases, even preceding them by years, with a progressive and degressive trajectory over the disease course (Krämer et al., 2015). Similarly, thalamic degeneration is a well-established feature of MS and represents one of the earliest neuroanatomical changes (Azevedo et al., 2018). Both structures have been shown to predict disease conversion and future disability (Clarke et al., 2022). Importantly, the thalamus serves as a critical hub for cortico-subcortical communication, and alterations in thalamo-cortical functional connectivity have been associated with disease progression and clinical severity in MS (Y. Liu et al., 2015; Schoonheim et al., 2022). Recent evidence has further linked thalamic atrophy to disruptions in thalamo-cortical white matter pathways and, notably, to alterations in microglial activity, including changes in cell density and morphology (Rodriguez-Mogeda et al., 2025). The thalamus has also been identified as a site of increased TSPO binding in MS patients compared to healthy individuals, as demonstrated by PET imaging studies (Laaksonen et al., 2023; Sucksdorff et al., 2020).

Regarding cLBP, the relatively modest performance observed for both disease classification and individual-level fingerprinting likely reflects the heterogeneous nature of the condition. Indeed, cLBP is an umbrella term that encompasses a broad range of underlying mechanisms. Our analyses revealed a substantially widespread involvement of both cortical and subcortical regions. However, the strongest contributors to disease classification were the accumbens area, the caudal and rostral anterior cingulate cortex (ACC), and the Banks of the superior temporal sulcus. The rostral ACC, as well as the lateral temporal cortex are part of the Default-Mode Network, a network that is highly implicated in the pathophysiology of chronic pain, including cLBP (Albrecht et al., 2021; Baliki et al., 2014; Loggia et al., 2013). The ACC represents a key hub within cortico-limbic circuits critically involved in the affective-motivational dimension and chronification of pain (Yang & Chang, 2019). Remarkably, the nucleus accumbens, which emerged as top contributor in the classification, is a key hub in pain processing and its alterations have been implicated in the transition to chronic pain (Chang et al., 2014; Harris & Peng, 2020; Makary et al., 2020). Evidence from TSPO PET imaging studies has demonstrated increased microglial activation in cLBP, particularly within the paracentral lobule, postcentral gyrus, and thalamus (Loggia et al., 2015). Although the main hubs identified by our network-based analysis did not directly overlap with these established neuroinflammatory regions, this discrepancy suggests that the computed network features may capture broader disease-related alterations beyond localized inflammation, potentially reflecting structural and functional reorganization associated with the pathological processes.

In the case of SCZ, our work revealed prominent involvement of the striatum — specifically the putamen and pallidum — alongside frontal cortical areas. The striatum has long been implicated in the pathophysiology of psychosis, primarily due to its central role in dopaminergic and glutamatergic dysregulation (Howes et al., 2024). Elevated presynaptic dopamine synthesis capacity and abnormal dopamine release in the striatum are replicated findings in schizophrenia research and are thought to underlie positive psychotic symptoms such as hallucinations and delusions (McCutcheon et al., 2019). Additionally, disruptions in glutamatergic transmission within striatal circuits have been proposed to further exacerbate these dopaminergic abnormalities, creating a dysregulated cortico-striatal-thalamic loop (Howes et al., 2015; McCutcheon et al., 2020). Numerous studies also report structural and functional abnormalities in frontal cortical regions in schizophrenia, including some that have associated frontal abnormalities with cognitive deficits and negative symptoms (DeLisi et al., 2006; Mubarik & Tohid, 2016). Meta-analyses of PET studies have found evidence for altered TSPO in frontal cortex in schizophrenia relative to controls, albeit the direction of group differences may depend on the outcome measure used (Marques et al., 2019). Our findings extend these prior findings and other evidence for glial cell abnormalities in schizophrenia to show that the inter-regional similarity of TSPO measures also differentiates people with schizophrenia from controls. These findings are consistent with the hypothesis that glial cell abnormalities lead to aberrant synaptic pruning in schizophrenia (Howes & Onwordi, 2023).

Regarding depression, our findings highlight a predominant involvement of frontal regions, particularly the caudal middle frontal area, the ACC, and the pars opercularis. Notably, these regions have all been shown to exhibit structural alterations in affected individuals (Leung et al., 2009; Schmaal et al., 2017; Suh et al., 2019). Alterations in frontal lobe volume and morphology, along with dysfunction of frontal-subcortical circuits, have been associated with the disorder (Zhang et al., 2018). Frontal regions play a key role in mood regulation and have been consistently implicated in depression (Goldin et al., 2008; Talbot & Cooper, 2006). Remarkably, the prefrontal cortex and ACC have been identified as regions exhibiting elevated microglial activation in individuals with depressive disorder (Holmes et al., 2018; Richards et al., 2018; J. J. Schubert et al., 2021; Setiawan et al., 2018).

Finally, our study explored the potential of individual-level patient fingerprinting. The analysis revealed above-chance performance levels, suggesting that condition-specific network signatures may transcend the group level, remaining detectable at the single-subject level. It is important to acknowledge, however, that the batch correction model likely influenced patient fingerprinting performance, potentially leading to overestimation for groups such as DEP and cLBP, which were the only diagnostic categories within their respective batches. Nevertheless, the good segregation achieved for MS patients, despite originating from different scanners and acquisition protocols, provides further evidence supporting the value of this approach for patient-level fingerprinting. Moreover, uncorrected graph elements from the [^11^C]-PBR28 KCL/ICL dataset demonstrated good individual fingerprinting capacity, with performance consistently above chance. Particularly noteworthy are the stable performances observed for TBI patients, who, despite being intrinsically heterogeneous, exhibited remarkably similar molecular network patterns. Importantly, these findings underscore the intrinsic value that network-derived representations could provide in neuroimaging for individual-level fingerprinting and precision medicine. The specificity of PET signal, combined with the inherently multivariate nature of network-based approaches, enables the detection of subject-specific patterns that may extend beyond the capabilities of traditional univariate neuroimaging analyses. This potential attests the robustness and translational relevance of this and comparable frameworks for delineating individualized signatures, in line with previous evidence across distinct disease contexts (Severino, Peretti, et al., 2025)

We acknowledge that the proposed methodology has some limitations. First, our selection of meaningful TSPO PET parameters to construct regional feature vectors was guided by previous research (Maccioni et al., 2025) and is therefore subject to the limitations of that original study. In particular, the parameter selection was optimized only for [^11^C]-PBR28 and applied without tailored calibration for [^11^C]-PK11195 and [^18^F]-DPA714. Additionally, our estimation of the *K*_*1*_ parameter, using a blood-free approach, was affected by the inherent limitations of the IDIF methodology (Maccioni et al., 2024; Volpi et al., 2023). Moreover, the retrospective nature of the study led to the inclusion of highly heterogeneous data. TSPO similarity matrices from healthy controls across different sites and scanners exhibited consistent spatial patterns, demonstrating the robustness of the methodology in handling heterogeneity. Nevertheless, the matrices remained sensitive to batch effects, likely reflecting differences in scanner models, image reconstruction methods, and acquisition protocols. Variations in acquisition protocols and tracer administration across PET centres can subtly alter tracer kinetics. Consequently, even after interpolating and standardizing TACs, signals may correspond to slightly different physiological states, affecting inter-regional distances. Differences in image reconstruction and scanner resolution likely further influence similarity calculation, but modelling these effects was not possible due to the lack of access to raw data. In addition, biological and experimental factors also influenced inter-regional similarities. Males exhibited higher similarity than females, consistent with sex-dependent differences in tracer delivery and binding (Barzon et al., 2025; Laaksonen et al., 2024; Tuisku et al., 2019). MABs showed higher global similarity then HABs, reflecting their greater proportion of non-specific binding and resulting in flatter spatial signal distributions. Conversely, similarity decreased with age, in line with increased heterogeneity of TSPO signal and loss of inter-regional coherence during aging (Farras-Permanyer et al., 2019; Fjell et al., 2016). Finally, increasing normalized dose was associated with higher inter-regional similarity for [^11^C]-PBR28 and [^11^C]-PK11195, likely reflecting progressive binding site saturation and signal homogenization, whereas for [^11^F]-DPA714, higher doses were associated with a slight reduction in similarity. To account for these sources of variability, harmonization of inter-regional similarities was performed using regression models that assumed the effects of technical and biological variables to be linear. However, non-linear effects may also be present. More complex harmonization approaches were deemed unfeasible due to the limited overall sample size, particularly the small number of scans from certain sites. Nevertheless, an advantage of the linear regression model lies in the ease of estimating coefficients using only healthy individuals, allowing for the correction of confounding factors while preserving disease-related variability. Future investigations applying network-based methodologies within a controlled setting, where healthy and multiple disease populations are acquired under standardized conditions at the same site, are warranted to further validate their potential for both group-and individual-level fingerprinting. As a sensitivity analysis, we examined site-specific classifiers trained on patients and their matched healthy controls without applying the harmonization procedure. All models performed above chance level, with β coefficients showing significant correlations with those of the full harmonized model across all diseases (Kendall’s tau, all P < 0.001; see **Supplementary Materials S.3** for full details). These results suggest that the main findings are robust to the confound correction strategy, although the full harmonized models remain more reliable, due to the larger training sample size, which yields more stable and robust estimates of the model coefficients.

A further limitation arises intrinsically from TSPO PET imaging, particularly concerning the biological interpretation of regional TSPO density as a marker of neuroinflammation. Although widely used across clinical domains, TSPO lacks cellular specificity, being expressed in microglia but also astrocytes, some neurons, endothelial cells, and infiltrating immune cells (Vicente-Rodríguez et al., 2021; Wijesinghe et al., 2025). However, recent studies have suggested that, although TSPO overexpression does not always reflect proinflammatory microglial activation, regional TSPO elevations reliably correspond to increased densities of glial and immune cells (Nutma et al., 2023). Supporting this, post-mortem evidence shows positive correlations between ante-mortem TSPO PET binding and microglial TSPO levels, as well as CD68 + phagocytic microglia (Wijesinghe et al., 2025). Overall, previous findings indicate that TSPO PET offers a meaningful measure of neuroinflammatory load. Despite concerns regarding cellular specificity, our method leverages TSPO PET signals to generate robust neuroinflammatory fingerprints and to potentially reveal the spatial specificity of TSPO PET to different inflammatory conditions, in line with the expected alterations associated with these diseases. These demonstrated that beyond the magnitude of brain TSPO binding increases, alterations in the topological pattern of TSPO expression and in the network of inter-regional relationships of TSPO PET signal that could reflect disease-specific neuroinflammatory profiles. Importantly, the framework is potentially translatable to future molecular targets for neuroinflammation and beyond, providing a versatile tool for both experimental and clinical investigations.

## Materials & Methods

### Study participants and image acquisition

The data analyzed in this work were gathered from *King’s College London (KCL), Imperial College London (ICL), Athinoula A. Martinos Center for Biomedical Imaging, Massachusetts General Hospital (MGH), Paris Brain Institute of Sorbonne University (ICM)*, and *University of British Columbia (UBC)* historical databases, including PET imaging data collected with three different TSPO radiotracers ([^11^C]-PBR28, [^18^F]-DPA714, and [^11^C]-PK11195). Due to the influence of the TSPO gene rs6971 polymorphism on radiotracer binding affinity (Owen et al., 2012), all participants acquired with [^11^C]-PBR28 or [^18^F]-DPA714 were genotyped prior to imaging. Only HABs and MABs were included in the study. A comprehensive summary of the participants’ characteristics is provided in Table 3. All procedures performed in studies involving human participants were in accordance with the ethical standards of the institutional and/or national research committee and with the 1964 Helsinki declaration and its later amendments or comparable ethical standards. Written informed consent was obtained from all individual participants included in the studies.

### [C]-PBR28 datasets

#### KCL/ICL dataset

The dataset consists of the aggregation of 118 dynamic [^11^C]-PBR28 PET scans from different imaging studies conducted at either at *KCL* and *ICL*, all acquired from the same facility and using the same imaging protocol. Specifically, dynamic scans were collected from 52 HCs, 15 individuals diagnosed with schizophrenia (SCZ), including 7 who underwent both a baseline and a blocking scan with XBD173 (Marques et al., 2021; Veronese et al., 2018), 14 participants with moderate-to-severe traumatic brain injury (TBI), and 5 patients with Alzheimer’s disease (AD), who also completed retest scans. Full details regarding participant inclusion criteria and imaging procedures are available in the original publications (Bloomfield et al., 2016; Dahoun et al., 2019; Nair et al., 2016; Scott et al., 2018). In brief, all imaging sessions started with a low-dose computed tomography (CT) scan for attenuation and scatter correction, performed on a Siemens Biograph TruePoint PET/CT scanner (Siemens Medical Systems, Germany). This was followed by a 90-minute dynamic PET scan initiated immediately after a bolus injection of [^11^C]-PBR28 (injected dose: 332.0 ± 26.9 MBq). The PET data were divided into 26 time frames (8×15s, 3×1 min, 5×2 min, 5×5 min, and 5×10 min), reconstructed using filtered back projection combined with 5 mm isotropic Gaussian smoothing, and corrected for random noise, attenuation, and scatter. Additionally, T1-weighted structural MR images were acquired using a Siemens 3-T scanner, either a Tim Trio or MAGNETOM Verio model.

#### MGH dataset

This dataset comprises a total of 94 [^11^C]-PBR28 PET scans acquired at *MGH*, as part of multiple imaging studies (Albrecht et al., 2019; Alshelh et al., 2020; Morrissey et al., 2023; Torrado-Carvajal et al., 2021). The first subset of the data, including 27 HCs and 21 patients with chronic low back pain (cLBP), was collected using a hybrid PET-MR system consisting of an avalanche photodiode-based PET scanner integrated with a Siemens 3T Tim Trio MRI scanner (Bay 6). Following a 90-minute dynamic PET acquisition initiated immediately after the bolus injection of [^11^C]-PBR28 (injected dose: 450.5 ± 63.0 MBq), data were reconstructed using the manufacturer’s implementation of the 3D Ordinary Poisson Ordered-Subset Expectation Maximization (OP-OSEM) algorithm. The reconstructed dataset was then segmented into 28 temporal frames with the following durations: 8×10s, 3×20s, 2×30s, 1×1min, 1×2 min, 1×3 min, 8×5 min, and 4×10 min. Prior to radiotracer injection, a multi-echo MPRAGE structural MRI was acquired to enable anatomical localization and to generate attenuation correction maps for PET image reconstruction.

A second subset of data, including 26 HCs and 20 cLBP patients, was collected at MGH on a Siemens Biograph mMR whole-body PET-MR (Bay 7). Dynamic PET data were acquired over a 90-minute period following bolus injection of [^11^C]-PBR28 (injected dose: 525.5 ± 52.9 MBq). The acquired data were divided into 28 temporal frames (duration: 8×10s, 3×20s, 2×30s, 1×1min, 1×2min, 1×3 min, 8×5 min, and 4×10 min). Image reconstruction was performed using the Ordered Subset Expectation Maximization (OSEM) algorithm, employing 4 iterations, 21 subsets, and a Gaussian smoothing filter with a 3 mm FWHM. T1-weighted MR images were acquired for anatomical reference and for the generation of attenuation correction maps. Comprehensive information regarding participant eligibility and imaging procedures can be found in the original publications.

#### UBC dataset

This dataset comprises 21 [^11^C]-PBR28 PET scans from 11 HCs and 10 individuals with SCZ, acquired with a GE SIGNA PET-MR scanner at *UBC*. Participants underwent a 90-minute PET acquisition following an intravenous bolus injection of [^11^C]-PBR28 (injected dose: 565.4 ± 9.0 MBq). PET data were binned into 25 temporal frames with the following durations: 1×20 s, 2×10 s, 4×5 s, 3×1 min, 3×2 min, 8×5 min, and 4×10 min and subsequently reconstructed using PSF-HYPR4D-K-TOFOSEM (Cheng et al., 2022). For anatomical localization and MRI-guided processing, all participants also underwent a high-resolution 3D T1-weighted MPRAGE sequence.

### [F]-DPA714 datasets

#### KCL dataset

The dataset includes 101 [^18^F]-DPA714 PET scans collected within the *Inflammatory Response In Schizophrenia (IRIS)*, carried out at the *Institute of Psychiatry, Psychology & Neuroscience, King’s College London* (https://clinicaltrials.gov/study/NCT03093064). The dataset includes 39 HCs and 62 patients with first-episode psychosis (FEP) who remained symptomatic despite antipsychotic treatment. Each participant underwent a 60-minute dynamic PET acquisition following a bolus injection of [18F]-DPA714 (injected dose: 187.1 ± 6.5 MBq), performed on a 3 T SIEMENS Biograph mMR PET-MR scanner. The PET data were reconstructed into 26 frames with the following timing sequence: 1×60 s, 8×15 s, 3×1 min, 5×2 min, and 9×5 min. Additionally, all subjects received a baseline T1-weighted structural MRI scan (MPRAGE sequence) acquired on the same scanner.

#### ICM dataset

This dataset comprises a total of 118 [^18^F]-DPA714 PET scans acquired at ICM as part of multiple imaging studies. The first subset includes scans from 14 healthy controls (HCs), 7 of whom underwent a retest scan within a period of 82 to 321 days following the initial acquisition, and 38 patients with multiple sclerosis (MS), all acquired using a GE SIGNA PET-MR scanner at SHFJ-CEA in Orsay and at Pitie Salpetriere Hospital. PET data were acquired over a 90-minute period following intravenous bolus injection of [^18^F]-DPA714 (injected dose: 222.7 ± 23.4 MBq). Image reconstruction was performed using a 3D Ordinary Poisson Ordered Subset Expectation Maximization (OP-OSEM) algorithm with integrated point spread function (PSF) modelling. The reconstructed data were divided into 33 temporal frames with the following durations: 9×20 s, 3×1 min, 7×2 min, and 14×5 min. In addition, all participants underwent a high-resolution 3D T1-weighted MPRAGE MRI sequence for anatomical reference.

A second subset consists of 15 HCs, including 8 who completed a retest scan within a period of 5 to 67 days following the initial acquisition, and 36 MS patients. For each participant, [^18^F]-DPA714 was administered intravenously as a slow bolus injection over a 1-minute period (injected dose: 196.3 ± 15.2 MBq). Dynamic PET data were then acquired on a High-Resolution Research Tomograph (HRRT, Siemens, Knoxville, TN, USA) at SHFJ-CEA in Orsay, featuring a transaxial field of view (FOV) of 31.2 cm and an axial FOV of 25.5 cm. PET images underwent corrections for attenuation, random events, and scatter, and were reconstructed using a 3D ordered-subset expectation maximization algorithm based on an Ordinary Poisson (OP-OSEM) statistical model. The resulting data were divided into 27 temporal frames (6×1 min, 7×2 min, and 14×5 min) A 3D Gaussian smoothing kernel with a 2 mm full width at half maximum (FWHM) was applied to account for the system’s point-spread function. Additionally, each subject underwent a T1-weighted MPRAGE MRI scan, acquired either on a Philips Achieva 1.5 T system (Best, The Netherlands) or a Siemens Trio 3 T system (Erlangen, Germany).

### [^11^C]-PK11195 dataset

#### KCL dataset

The dataset comprises 76 dynamic PET scans with [^11^C]-PK11195, collected as part of the Biomarkers in Depression study (BIODEP). It includes scans from 51 individuals with mild to moderate depression (DEP) and 25 matched healthy control (HC) participants. Further information on subject recruitment and clinical assessments can be found in previous studies (Turkheimer et al., 2021; Schubert et al., 2021). All participants underwent a 60-minute dynamic PET scan using a GE SIGNA PET/MR scanner (GE Healthcare, Waukesha, USA) following an intravenous bolus injection of [^11^C]-PK11195 (injected dose: 366 ± 51 MBq). The data were divided into 17 frames (4×15s, 4×1min, 7×5min, 2×10min) and reconstructed using a multi-subject atlas approach optimized for the MRI brain coil. Standard corrections for scatter, random events, and dead time were applied using the GE scanner software. Additionally, each subject underwent a high-resolution T1-weighted structural brain MRI (BRAVO) during the PET acquisition.

Acronyms: KCL, King’s College London; ICL, Imperial College London; MGH, Massachusetts General Hospital; UBC, University of British Columbia; ICM, Paris Brain Institute; HC, healthy control; SCZ, schizophrenia; AD, Alzheimer’s disease; TBI, traumatic brain injury; cLBP, chronic low back pain; FEP, first-episode psychosis; MS, multiple sclerosis; DEP, mild-to-moderate depression.

##### Data preprocessing and TACs extraction

Data were pre-processed at each neuroimaging centre using various combinations of in-house scripts and established analysis software, including the FMRIB Software Library (FSL, http://www.fmrib.ox.ac.uk/fsl), Statistical Parametric Mapping (SPM, http://www.fil.ion.ucl.ac.uk/spm), FreeSurfer (https://surfer.nmr.mgh.harvard.edu), and MIAKAT (Gunn et al., 2016). The pre-processing pipeline for all scans comprised motion correction of dynamic PET data, generation of integral PET images, extraction of brain and grey matter masks from structural T1-weighted MRI, and registration of these masks to each subject’s native PET space. Additionally, the FreeSurfer APARC + ASEG neuroanatomical parcellation, which includes cortical regions from the Desikan-Killiany atlas and subcortical areas from the ASEG segmentation, was co-registered to the individual PET images. For subsequent analyses, a total of 87 ROIs were selected: 68 bilateral cortical regions, 16 subcortical regions, the bilateral cerebellar cortex, and the brainstem. Finally, ROIs mean regional time-activity curves (TACs) were computed for each subject. For MS patients, the preprocessing pipeline incorporates a lesion inpainting step to enhance atlas coregistration and mitigate structural artifacts arising from lesions (see (Chard et al., 2010) for further details).

##### Networks construction

The present method was inspired by recent molecular connectivity approaches based on Euclidean distance metrics applied to TACs (Volpi et al., 2023). However, rather than relying on full dynamic PET signals, our approach focuses on a reduced set of biologically meaningful regional parameters. For each scan, regional TACs were interpolated onto a common sparse time grid. Then, for each region, a feature vector was constructed including standardized uptake values (SUV) at three representative time points (1.25, 13.5, and 50 minutes post-injection) and the regional *K₁* parameter estimated with a single irreversible compartment model, employing an image-derived input function (IDIF) (Barzon et al., 2025; Maccioni et al., 2024). These parameters were selected based on a recently proposed methodology for TSPO PET quantification (Maccioni et al., 2025), having been identified by a logistic regression model as the most informative for differentiating regions with high and low TSPO expression, thereby reflecting the inflammatory status of cerebral tissue. To account for inter-subject variability and ensure that each feature contributed equally to the calculation of the Euclidean distance, all features were standardized within each tracer group. This was achieved by applying a robust z-score transformation, calculated as the difference between each value and the group median, divided by the median absolute deviation. Individual TSPO PET similarity matrices were derived by computing, for each pair of regions, the normalized inverse Euclidean similarity between regional feature vectors, defined as

$$S_{ij}=\frac{1}{1\;+\;d_{ij}}$$

where $d_{ij}$ is the Euclidean distance between the feature vectors of ROI i and ROI j, computed as

$$d_{ij}=\sqrt{\sum_{k=1}^{n}\;\;{\left(x_{ik}-x_{jk}\right)}^{2}}$$

with $x_{ik}$ representing the standardized value of feature k within the feature vector of ROI i, and n being the total number of features in the vector. Higher values of $S_{ij}$ indicate greater similarity between regional molecular profiles.

To assess the inter-subject consistency of network patterns, pairwise Spearman’s correlations were computed between the upper triangular elements of each healthy subject’s resulting similarity matrix.

##### Evaluation of biomarker biological specificity and reproducibility

The specificity of TSPO similarity matrices for the TSPO molecular target was assessed through a retrospective analysis of data acquired before and after pharmacological partial blockade of the target using the ligand XBD173 in a cohort of 7 SCZ patients (Veronese et al., 2018). Specifically, the median of the elements in the upper triangular portion of the connectivity matrices was compared before and after blocking using a one-tailed Wilcoxon signed-rank test, to test the hypothesis that target blockade would result in a flatter and more homogeneous signal distribution across the brain, thereby increasing the average similarity values between regions. One subject was excluded prior to the evaluation due to unexplainable variability in the baseline scan (Marques et al., 2021).

The reproducibility of TSPO similarity matrices was evaluated in cohorts for which test-retest scans were available, specifically 5 patients with AD acquired with [^11^C]-PBR28 at *KCL* and 15 HCs scanned with [^18^F]-DPA714 at *ICM*. Spearman’s correlation was computed between the corresponding elements of the upper triangular portions of the test and retest similarity matrices. Additionally, edgewise intraclass correlation coefficients (ICCs; type ICC(3,1), two-way mixed-effects model, single measurement) were computed for each element of the similarity matrices across test and retest sessions. Finally, a fingerprinting analysis (J. Liu et al., 2018) was performed by testing the ability to correctly identify, for each test scan, its corresponding retest scan as the most similar in terms of correlation between TSPO similarity matrices.

##### Evaluation of technical and biological effects on TSPO similarity matrices

A set of statistical and machine learning methods was applied to healthy individuals to assess the potential influence of various factors on TSPO network features. Specifically, the effects of batch (a combination of scanner, site, and acquisition protocol), TSPO genotype, sex, age, and radiotracer DW were evaluated.

To investigate batch effects, data were first regressed for potential confounders, including sex, TSPO genotype, age, and DW, by applying a linear model to each edge value and retaining the residuals. The UMAP (McInnes et al., 2018) was then performed for visual inspection of potential clustering. Subsequently, three machine learning classifiers—logistic regression with elastic net regularization, k-nearest neighbors, and support vector machines (with linear, polynomial, and radial basis function kernels)—were trained to predict batch labels within each tracer group. Data were split 70/30 into training and testing sets, with all features standardized, followed by 10-fold cross-validation (CV) and grid search for hyperparameter optimization. Balanced accuracy was used as scoring metric during CV and for model evaluation on the test set. To estimate the 95% confidence interval of test-set performance, 1000 bootstrap iterations were conducted.

The same framework was adopted to assess the effects of sex and TSPO genotype. Following batch effect regression, binary classification models were trained within each tracer cohort using the same set of classifiers and procedures.

Additionally, to quantify the effects of all technical and biological variables on TSPO networks, tracer-specific multiple linear regression models were applied to each edge value. For each regressor, the distribution of standardized beta coefficients across all edges was examined, and Cohen’s *d* was computed to determine whether the overall effect of a given variable on similarity values was predominantly positive or negative, and thus whether the beta coefficients were, on average, significantly greater or less than zero.

##### Disease classification

Disease classification analyses were conducted using a one-vs-all approach, in which each diagnostic group was classified against all HC scans acquired with the same tracer. An exception was made for SCZ patients, who were pooled across tracers (including also individuals with FEP) and classified against a combined HC group from [^18^F]-DPA714 and [^11^C]-PBR28 cohorts. For each classification task, the dataset was randomly split into 70% for training and 30% for testing. The effects of batch, sex, TSPO genotype, age, and radiotracer dose-to-weight ratio were regressed out from the network edge values using the previously described linear models. To prevent the removal of disease-related variance and avoid data leakage, confound correction models were fitted exclusively on the HCs in the training set and then applied to the entire dataset. Afterward, features were z-scored to allow direct comparison of model coefficients. Model training involved a stratified 10-fold CV on the training set, performed 10 times to enhance reliability. When class sizes were limited, stratified *k*-fold CV was employed, ensuring that each fold contained at least one individual from the positive class.

For this task, we selected a logistic regression model with L1 regularization for its interpretability and its ability to enable post-hoc identification of relevant features for classification through the examination of non-zero coefficients, while offering a fully data-driven approach that does not require prior feature selection. Model performance was evaluated using the Average Precision (AP) score, which corresponds to the area under the precision-recall curve. AP was chosen as it is threshold-independent and particularly well-suited for handling class imbalance, which was characteristic of the datasets. AP was used consistently as the scoring metric during CV for model selection and as the primary evaluation metric on the test set. Receiver operating characteristic area under the curve (ROC AUC) was also computed on the test set. The 95% confidence intervals for AP and ROC AUC were estimated using 1,000 bootstrap iterations.

To interpret model outputs and identify the most discriminative brain regions, regional importance scores were computed. Specifically, for each ROI, the importance score was quantified as the sum of the absolute values of the logistic regression coefficients associated with all network edges connected to that ROI. Moreover, to assess the distinctiveness of the network profiles associated with each disease, pairwise Kendall’s tau correlations were computed between the beta coefficients of the logistic regression models from each classification task. This analysis quantified the degree of overlap in feature importance patterns across diseases, providing a measure of how similar or distinct the discriminative network features were between different pathological conditions.

Finally, a multiclass logistic regression classifier with L1 regularization was trained using data from all tracers and centres to discriminate between the different disease groups. The same confound correction and training procedure described above was applied. Model performance was assessed on the test set using balanced accuracy, with 95% confidence intervals estimated as previously described.

##### Individual-level patient fingerprinting

Lastly, we investigated the feasibility of individual patient fingerprinting based on TSPO network profiles. To account for batch effects, a linear regression model was trained on healthy controls and subsequently applied to all subjects to remove the influence of batch, sex, TSPO genotype, age, and radiotracer dose-to-weight ratio. Pairwise Spearman’s correlations were then computed between the upper triangular elements of each subject’s TSPO similarity matrix. Each patient was assigned to the diagnostic group of the subject with whom they shared the highest correlation. A fingerprinting confusion matrix was generated to summarize classification performance, from which balanced accuracy and per-group recall were derived.

Given the potential risk that batch correction might inflate apparent disease-related effects—potentially due to the collinearity between disease status and batch membership— we repeated the fingerprinting analysis using the original, uncorrected network features within the [^11^C]-PBR28 dataset from KCL, as this batch included more than one clinical group, namely SCZ and TBI. This sensitivity analysis ensured that fingerprinting performance was not artificially driven by overcorrection artifacts.

##### Software implementation

All network construction and statistical analyses were implemented in MATLAB R2022b (MathWorks). Machine learning models, including classification tasks and multiple linear regressions, were developed in Python (version 3.8.13) using the scikit-learn library (version 1.3.2) (Pedregosa et al., 2011).

## Supplementary Material

Supplementary Files

This is a list of supplementary files associated with this preprint. Click to download.

• SupplementaryTSPOnetworksPREPRINT.pdf

## Figures and Tables

**Table 1:** Classification performance for batch, sex, and TSPO genotype prediction.

Tracer	Target variable	Best model	Balanced accuracy
[^11^C]-PBR28	Batch	KNN	0.89 [0.79, 0.98]
	Sex	KNN	0.63 [0.49, 0.77]
	Genotype	KNN	0.51 [0.35, 0.68]
[^18^F]-DPA714	Batch	KNN	0.94 [0.86, 1.00]
	Sex	LR	0.71 [0.52, 0.91]
	Genotype	SVM	0.68 [0.45, 0.90]
[^11^C]-PK11195	Sex	KNN	0.75 [0.38, 1.00]

*Best model* refers to the classifier achieving the highest balanced accuracy on the test set. Classifiers include k-nearest neighbour (KNN), logistic regression (LR), and support vector machine (SVM) with linear kernel. Balanced accuracy on the test set is reported alongside corresponding 95% confidence intervals from 1,000 bootstrap iterations.

**Table 2:** Disease classification performance.

Condition	Test set AP	AP chance level	Test set ROC AUC
TBI	1.00 [1.00, 1.00]	0.11	1.00 [1.00, 1.00]
DEP	0.96 [0.89, 1.00]	0.65	0.92 [0.78, 1.00]
MS	0.81 [0.66, 0.97]	0.52	0.85 [0.71, 0.96]
cLBP	0.51 [0.37, 0.79]	0.24	0.81 [0.68, 0.91]
SCZ	0.70 [0.57, 0.84]	0.47	0.74 [0.61, 0.87]

For each condition test set AP, AP chance level, and test-set ROC AUC are reported, with 95% confidence intervals from 1,000 bootstrap iterations. Acronyms: AP, average precision; ROC AUC, receiver operating characteristic area under the curve; TBI, traumatic brain injury; DEP, mild-to-moderate depression; MS, multiple sclerosis; cLBP, chronic low back pain; SCZ, schizophrenia

**Table 3:** Demographic and clinical characteristics of participants

Radiotracer	Site	Scanner	Group	Age (y)	Sex (M/F)	Affinity (HAB/MAB)
[^11^C]-PBR28	KCL/ICL	Siemens Biograph TruePoint PET-CT	HC (n = 72)	32 ± 13	50/22	50/22
			SCZ (n = 15) + 7 blocking	46 ± 10	12/3	13/2
			AD (n = 5) + 5 retest	82 ± 4	4/1	5/0
			TBI (n = 14)	42 ± 14	12/2	9/5
	MGH (Bay 6)	PET-MR: PET within Siemens 3T Tim Trio MR	HC (n = 27)	47 ± 13	13/14	17/10
			cLBP (n = 21)	43 ± 14	10/11	17/4
	MGH (Bay 7)	Siemens Biograph mMR whole-body PET-MR	HC (n = 26)	55 ± 15	12/14	13/13
			cLBP (n = 20)	47 ± 17	9/11	11/9
	UBC	GE SIGNA PET-MR	HC (n = 11)	35 ± 10	3/8	8/3
			SCZ (n = 10)	35 ± 8	2/8	5/5
[^18^F]-DPA714	KCL	Siemens Biograph mMR PET-MR	HC (n = 39)	29 ± 8	21/18	29/10
			FEP (n = 62)	28 ± 8	41/21	41/21
	ICM (PET-MR)	GE SIGNA PET-MR	HC (n = 14) + 7 retest	43 ± 13	9/5	8/6
			MS (n = 38)	46 ± 10	16/22	18/20
	ICM (HRRT)	Siemens HRRT	HC (n = 15) + 8 retest	43 ± 13	6/9	7/8
			MS (n = 36)	48 ± 11	15/21	21/15
[^11^C]-PK11195	KCL	GE SIGNA PET-MR	HC (n = 25)	37 ± 8	11/14	/
			DEP (n = 51)	36 ± 7	15/36	/

Acronyms: KCL, King’s College London; ICL, Imperial College London; MGH, Massachusetts General Hospital; UBC, University of British Columbia; ICM, Paris Brain Institute; HC, healthy control; SCZ, schizophrenia; AD, Alzheimer’s disease; TBI, traumatic brain injury; cLBP, chronic low back pain; FEP, first-episode psychosis; MS, multiple sclerosis; DEP, mild-to-moderate depression.

## Data Availability

Restrictions apply to the availability of the data analyzed for this study which were used under license for the current study and are not publicly available. Data are available upon reasonable request to the corresponding authors.
